# Non-disease specific patient-reported outcome measures of health-related quality of life in juvenile idiopathic arthritis: a systematic review of current research and practice

**DOI:** 10.1007/s00296-021-05077-x

**Published:** 2021-12-31

**Authors:** Justyna Młyńczyk, Paweł Abramowicz, Maciej K. Stawicki, Jerzy Konstantynowicz

**Affiliations:** Department of Pediatrics, Rheumatology, Immunology, and Metabolic Bone Diseases, University Children’s Hospital, Medical University of Bialystok, Waszyngtona Street 17, 15-274 Bialystok, Poland

**Keywords:** Juvenile arthritis, Health-related quality of life, Patient-reported outcome measures, Children

## Abstract

Juvenile idiopathic arthritis (JIA), as a chronic condition, is associated with symptoms negatively impacting health-related quality of life (HRQL). Regarding growing interest in the implementation of the patient-reported outcome measures (PROMs), we aimed to review the non-disease specific PROMs addressing HRQL assessment, potentially useful in the clinical care of JIA and daily practice. A systematic literature search was conducted using MEDLINE/PubMed, Google Scholar, Scopus and Embase databases (1990 to 2021), with a focus on the recent 5-years period. Entry keywords included the terms: “children”, “adolescents”, “JIA”, “chronic diseases”, “HRQL”, “PROMs” and wordings for the specific tools. Several available PROMs intended to measure HRQL, non-specific to JIA, were identified. The presented outcomes differed in psychometric properties, yet all were feasible in assessing HRQL in healthy children and those with chronic diseases. Both EQ-5D-Y and PedsQL have already been tested in JIA, showing relevant reliability, validity, and similar efficiency as disease-specific measurements. For PROMIS® PGH-7 and PGH-7 + 2, such validation and cross-cultural adaptation need to be performed. Considering the future directions in pediatric rheumatology, the large-scale implementation of PROMIS® PGH-7 and PGH-7 + 2 in JIA offers a particularly valuable opportunity. The PROMs reflect the patient perception of the chronic disease and allow to understand child’s opinions. The PROMs may provide an important element of the holistic medical care of patients with JIA and a standardized tool for clinical outcomes, monitoring disease severity and response to treatment.

**Supplementary Information:**

The online version contains supplementary material available at 10.1007/s00296-021-05077-x.

## Introduction

In the past decades, the Quality of Life (QoL) has become a significant objective of medical research and an important component of public health. Furthermore, the patient perspective has gained a new role in contemporary health care worldwide, particularly regarding chronic diseases. The meaning and perception of QoL can be interpreted in various ways [1], nonetheless, according to the World Health Organization guidelines, QoL is defined as „an individual's perception of their position in life in the context of the culture and value systems in which they live and in relation to their goals, expectations, standards and concerns” [2].

A more specific term, health-related quality of life (HRQL), reflects the correlation between health and functioning of an individual [3]. HRQL outcomes are recognized as a credible rating of subjective health of adults [4], as well as that of children and adolescents [5]. Even though the understanding and interpretation of HRQL vary between individuals, causing thereby the assessment of the outcome a great challenge, some influential organizations like the National Institute for Health and Care Excellence (NICE) recommend HRQL measurements in clinical care and trials [6, 7].

One of the newly implemented methods allowing evaluation of HRQL are patient-reported outcome measures (PROMs). An increasing interest in using PROMs in clinical practice has been noted [8] whereas the PROMs have been shown as an important element of different areas of healthcare [9]. The PROMs are able to measure “patient’s health status that comes directly from the patient, without the interpretation of the patient’s responses”, according to the current definition [10], and provide the patient’s perspective and information that can only be given by patient himself [11]. So the core element is the direct and reliable response. They comprise a possibility to expand knowledge about patient’s functioning, identify problems affecting patient, improve treatment and care, and allow to construct health-care responsive to the patient [1, 12]. Importantly, PROMs may also be useful to indicate the correlation between healthcare interventions and patients’ HRQL [13].

HRQL is qualified as a crucial outcome of chronic health conditions [14, 15], e.g. diabetes, asthma, juvenile arthritis, and its improvement should be one of the main goals of patient care [16]. It is well known that chronic conditions predispose to lower HRQL, however, not only disease symptoms per se may implicate reduced HRQL, the duration of the disease is also relevant [17]. Other factors impacting HRQL are e.g. socioeconomic status, psychosocial behavior and support, and health behaviors [18]. In view of the abovementioned factors, subjective psychical or mental health complaints among patients with chronic conditions can result in deterioration of HRQL and should be definitely taken into consideration in patient care [19, 20].

Juvenile Idiopathic Arthritis (JIA) is one of the most prevalent chronic conditions of childhood, with the prevalence of 1 per 1,000 children, moreover, it is the most common pediatric rheumatic disease [21]. According to the classification criteria, JIA includes seven subtypes stratified by the number of involved joints, presence of systemic features, and additional markers as rheumatoid factor (RF) [22]. The subsets of JIA differ regarding the clinical picture, and also pathogenic background, i.e. etiopathogenesis and genetics, yet so all of them contribute to the development of disability and deterioration of HRQL to a great extent [23, 24].

A core feature of JIA is the inflammation of joints such as knees, ankles, hands, elbows, and/or wrists. Symptoms include swelling, pain, stiffness, and limited function of joints [25], with pain being the most commonly observed and disturbing ailment [26]. It is documented that children with a prolonged course of the disease may have modified pain sensitivity and perception [27], and reduced physical activity due to functional impairment [28, 29]. JIA can proceed also with extra-articular manifestations, e.g. fevers, erythematous rash, hepatosplenomegaly, enlarged lymph nodes, serositis, enthesitis [30] and not to mention uveitis, the most widely appearing extra-articular sign of JIA, potentially leading to vision loss [31]. Patients with JIA may experience deterioration of emotional and psychosocial functioning, given that anxiety and depressive symptoms are related to pain and decreased mobility [32]. Furthermore, JIA, as a chronic condition, is linked to increased use of healthcare services, taking into account frequent hospitalizations [33]. In summary, children and adolescents with JIA are at a higher risk of a lower HRQL due to the chronic nature of the disease, limited mobility, and the symptoms listed above [34].

Regarding the importance of HRQL assessment in the clinical care of patients with chronic diseases, implementation of HRQL, especially patient-reported, outcomes should be discussed more extensively [35]. In this report, we presented several currently available PROMs addressing HRQL measurements, which could be useful and may be potentially included in daily practice in JIA. We focused on the JIA-non-specific outcomes, given that HRQL JIA- specific measurements, e.g. JAMAR [36] or JAQQ [37] are commonly known and accepted. The state of the art suggests that development of the new methods allowing to assess of HRQL data from a child’s level in this condition is particularly needed.

## Methods and search strategy

**Fig. 1:**
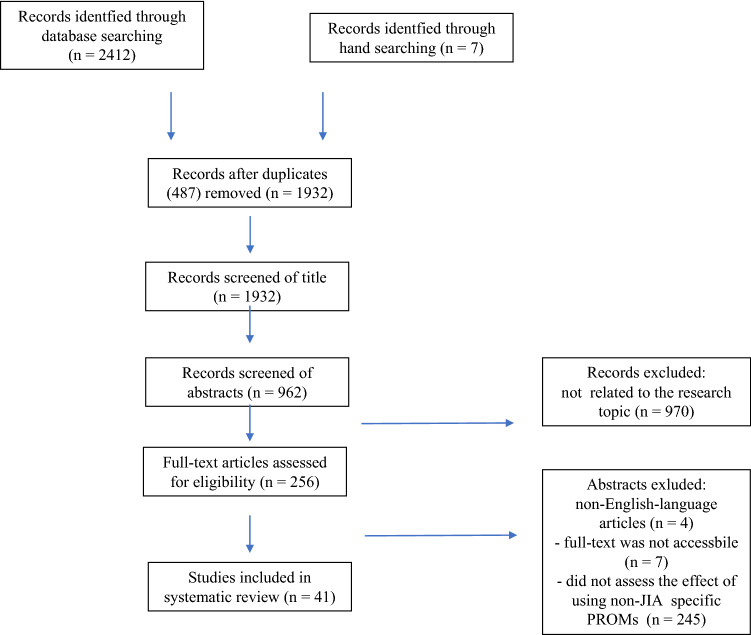
Flow diagram of database searching

We conducted a systematic literature search using Medline (via PubMed), Google Scholar, Scopus, and Embase electronic databases (through 1990 to 2021), focusing on the last 5-years period. Based on the MeSH thesaurus, we used entry key words for “children”, “adolescents”, “JIA”, “chronic diseases”, “HRQL”, “PROMs” and the available eligible names of the instruments applicable for its assessment. The search was limited to English-language articles, both systematic reviews, and original papers. All abstracts were examined, followed subsequently by full-text analysis and review, resulting in evaluation of univocity and trustworthiness of the study. Study protocol, including literature references search, article collection, eligibility, data extraction, and subsequent review and discussion, was based on current recommendations [38–40]. A total of 2412 published records were screened and, following standardized search process, finally 41 articles were included in the review (Fig. 1).

After comprising a list of HRQL outcome measures, we selected several for further and a more detailed review, all of them eventually permitting to obtain patient-reported data. We presented current information on the use of the measures: reliability, validity, practical applications, and psychometric properties. The summarized comparative data were presented in Table 1.

**Table 1:** Characteristics, practical applications, and psychometric properties of the health- related quality of life patient-reported outcomes measurements

Measure	Content/domains	Number of items + Response options	Methods of administration	Age of the respondents	Recall period	Language translations	Scoring	Relia- bility	Vali- dity	Used in JIA
PROMIS Pediatric global health 7	HRQL (general, physical, mental and social health)	7 items; 5-point ordinal scale	Paper or electronic, self- report or parent proxy- report	8–17 years for self- report and 5- 17 years for parent proxy report	–	English + 17 different language versions	Global health score scored with item- level calibrations; T-score metric with a mean = 50, SD = 10	Yes	Yes	No
PROMIS Pediatric global health 7 + 2	HRQL (general, physical, mental and social health) + pain interference and fatigue	9 items; 5-point ordinal scale	Paper or electronic, self- report or parent proxy- report	8–17 years for self-report and 5–17 years for parent proxy report	7 days for pain interference and fatigue item	English + 18 different language versions	Global health score scored with item- level calibrations + pain interference score + fatigue score; T-score metric with a mean = 50, SD = 10	–	–	No
EQ-5D-Y	HRQL (domains: mobility, self-care, usual activities, pain/discomfort, anxiety/depression) + visual analog scale	5 items; 3 levels of severity for each item	Paper or electronic, self- report or parent proxy- report	8–15 years for self-report and 4-15 years for parent proxy report	Present (TO- DAY)	English + over 50 different language versions	Health state, described by a 5-digit number (each of the digits presents the level of severity selected in the items); single summary number (index value)	Yes	Yes	Yes
PedsQL 4.0 Generic core scales	HRQL (domains: physical functioning, emotional functioning, social functioning, school functioning)	5–8 items per domain (23 in total)	Self-report or parent proxy- report	5–18 years for self-report and 2-18 years for parent proxy report, 5-point ordinal scale	one month, 7 days for an acute version	English + different language versions	Separate scales for each of the dimensions, and three summary scores (Total Scale Score, Physical Health Summary Score, Psychosocial Health Summary Score), reverse- scored from 0 to 100	Yes	Yes	Yes
PedsQL 4.0 SF15 Generic core scales	HRQL (domains: physical functioning, emotional functioning, social functioning, school functioning)	3–5 items per domain (15 items in total)	Self-report or parent proxy-report	5–18 years for self-report and 2-18 years for parent proxy report, 5-point ordinal scale	One month, 7 days for an acute version	English + different language versions	Separate scales for each of the dimensions, and three summary scores (Total Scale Score, Physical Health Summary Score, Psychosocial Health Summary Score), reverse-scored from 0 to 100	Yes	Yes	–
PedsQL 3.0 rheumatology module	HRQL (domains: pain and hurt, daily activities, treatment, worry, communication)	3–7 items per domain (22 in total)	Self-report or parent proxy-report	5–18 years for self-report and 2–18 years for parent proxy report, 5-point ordinal scale	One month	English + different language versions	Reverse- scored from 0 to 100	Yes	Yes	Yes

A comparison of different methods is shown

## Results

### EuroQoL (EQ-5D)

#### Content

The EuroQol’s EQ-5D standardized non-disease specific instruments were developed in 1990 to delineate and evaluate health-related quality of life [41]. The EuroQol measurements produce a generic cardinal index of health, aiming to assess the physical, mental, and social functioning of an individual [42], and are today widely recommended by several health technology assessment agencies for use in the cost-utility analysis [43].

Standard EQ-5D construction consists of a descriptive system questionnaire of five dimensions: 1- mobility, 2- self-care, 3- usual activities, 4- pain/discomfort and 5- anxiety/depression called EQ-SD self-classifier, and an EQ-VAS (visual analog system) vertical scale rating current health status with the endpoints 100 at the top of the scale and 0 at the bottom [44]. The EQ-5D instruments occur in two basic forms, the EQ-5D-3L version with three levels of severity in each of the five dimensions, and the EQ-5D-5Lversion with five levels of severity in each of the five dimensions [45]. Besides the increased number of severity levels the EQ-5D-5L form differs from the EQ-5D-3L in the changed label of the most severe option regarding the mobility dimension [Supplementary material].

In 2009, the EuroQol Group implemented the EQ-5D-Y, based on the EQ-5D-3L self- completed instrument suitable for children and adolescents. The EQ-5D-Y, as other EQ-5D measurements, contains the EQ-5D descriptive system questionnaire and the EQ-VAS. The EQ-5D-Y’s descriptive system is composed of five dimensions, listed as 1- mobility (walking about), 2- looking after myself, 3- doing usual activities (for example going to school, hobbies, sports, playing, doing things with family or friends), 4- having pain or discomfort and 5- feeling worried, sad or unhappy. As can be seen above, the headers of the dimensions were specified and adapted obviously to younger respondents to refine the comprehension of the text. Each of the five dimensions has assigned three levels of severity, referring to the present health state (TODAY) [46]. In contrast to the adult version EQ-5D-3L, the EQ-5D-Y has modified labeling of the most severe option in all of the dimensions, also the wording of the mildest option was transformed in the looking after myself dimension [Supplementary material]. In this article the EQ-5D-Y (with three levels of severity) has been detailed but, noticeably, a new version of the instrument with five severity levels, the EQ-5D-Y-5L, is under development [47–49]. The second component of the instrument, the EQ-5D-Y VAS, preserves standard vertical construction. Instructions for the EQ-VAS were considerably simplified and clarified to reassure a more child-friendly and appropriate approach.

#### Practical application

The EQ-5D-Y assessment is designed to be used for children and adolescents aged 8–15 years. For older children, 16-year-old and above, adult versions of EQ-5D instruments are usually recommended, while for children younger than 8 years (4–7 years), a parent proxy-reported measurement should rather be used. There are two proxy versions available: version 1, in which the caregiver rates the child’s HRQL in their (proxy’s) opinion, and version 2, in which the caregiver is asked to rate the HRQL from a child’s position (how a child would rate his/her HRQL). The EQ-5D-Y is obtainable in various formats (paper and digital on PDAs/smartphones/tablets/computers) and a number of translations (currently more than 50 self-complete language versions).

#### Scoring

The aim of the EQ-5D-Y is to indicate the respondent’s health state, numerically described by a 5-digit code. This 5-digit code emerges from combining 1-digit numbers corresponding to the level of severity selected in every dimension of the descriptive system questionnaire. In conclusion, there is a possibility to define in total 243 diverse health states. The health status can also be presented as a single summary number (index value) reflecting the qualitative assessment of health in compliance with the preferences of the general population of a given country/region (value sets). Index values facilitate calculating quality-adjusted life years (QALYs), which can be particularly useful in the cost-utility analysis [50].

#### Psychometric properties

The EQ-5D-Y’s feasibility, reliability, and validity were legitimated in the research conducted on German, Italian, South African, Spanish, and Swedish children and adolescents. The reported agreement in test–retest reliability ranged between 69.8 and 99.7%, with Kappa coefficients about 0.67. Validity was established by comparing children with a previously foreseen disparity in HRQL [51]. According to a study conducted on a representative sample of Canadian children, also the VAS-based index demonstrated logical consistency, with no statistically important disparity between the actual and predicted VAS values [52].

The instrument has been implemented in several well- designed studies of chronic conditions, e.g. asthma [53], diabetes type 1 [54–56], and JIA. Findings from a study conducted on 219 English-speaking children aged 8–15 years revealed that the EQ-5D-Y was valid in the determination of HRQL among pediatric patients with JIA. Moreover, the report demonstrated this generic instrument had similar efficiency as disease- specific measurements, contributing to a more productive JIA management [57].

### Pediatric quality of life inventory (PedsQL)

#### Content

The PedsQL, designed in 1999 by Varni et al., is a standardized generic instrument to measure HRQL in the pediatric population [58]. More specifically, this modular measurement system consists of the generic core scales and complementing them disease-specific modules, allowing assessment of HRQL across a broad spectrum of pediatric populations, including healthy children and those with chronic conditions. In 2002, the PedsQL 3.0 Rheumatology Module was developed, encompassing a specific module with a great scope of HRQL assessment across various dermatological conditions, connective tissue diseases, and musculoskeletal diseases [59].

The PedsQL 4.0 Generic Core Scales, self and parent proxy reported, in versions for children aged 5–18 years contain 23 items related to four major dimensions:1- physical functioning (8 items), 2- emotional functioning (5 items), 3- social functioning (5 items), and 4- school functioning (5 items), with suitable response options ranging from 0 (never) to 4 (almost always). In a self-reported version for younger children (5–7 years), a simplified, graphically shown as a happy-to-sad face 3-point scale is used. The parent proxy report for toddlers aged 2–4 years includes 21 items in the same four, listed above, dimensions. This form differs by limiting to 3 items in the school functioning dimension.

The PedsQL 4.0 Generic Core Scales are also available in shorter simplified forms: The PedsQL 4.0 SF15 Generic Core Scales, composed of 15 items derived from the original measurements. For all of the age-specific versions, the PedsQL SF15 comprises the same four dimensions followed by the same response options as in the original measurements.

The PedsQL 3.0 Rheumatology Module in versions (self and parent proxy reported) for children aged 8–18 years is composed of five dimensions containing 22 items in total, as listed: 1- pain and hurt (4 items), 2- daily activities (5 items), 3- treatment (7 items), 4- worry (3 items), 5-communication (3 items). Response options are 5-point scaled from 0 (never) to 4 (almost always). Both self and parent proxy reports for children aged 5–7 years comprise 20 items in the same five dimensions. Response options in the self-report are the same as in the age-adjusted PedsQL 4.0 Generic Core Scales version i.e. graphically shown 3-point scale. The parent proxy report for toddlers aged 2–4 years describes three dimensions: 1-pain and hurt (4 items), 2-daily activities (5 items), and 3-treatment (5 items).

The PedsQL 4.0 Generic Core Scales and the PedsQL SF15 are offered in a variant with a 1-month recall period, and in an acute variant with a recall period lasting 7 days. Such methodological flexibility may be useful in adjustment for disease or treatment duration. All of the PedsQL 3.0 Rheumatology Module versions are designed to achieve a one-month recall period.

#### Practical application

The PedsQL measurements are available in numerous language translations and can be administered as both self and parent proxy report in versions categorized as for younger children aged 5–7, children aged 8–12, adolescents aged 13–18 years, and as a parent proxy report for toddlers aged 2–4 years. The method is based on paper format, however, for the PedsQL 4.0 Generic Core Scales self-report for adolescents a convenient e-version is also accessible.

#### Scoring

The PedsQL 4.0 Generic Core Scales generate four all-encompassing scales, one for each of the dimensions, and three summary scores 1- Total Scale Score (23 items), 2- Physical Health Summary Score (8 items), and 3- Psychosocial Health Summary Score (15 items). The PedsQL SF15 produces, respectively four scales, one for each of the dimensions, and three summary scores 1-Total Scale Score (15 items), 2-Physical Health Summary Score (5 items), and 3-Psychosocial Health Summary Score (10 items). The measurements, containing the PedsQL 3.0 Rheumatology Module, using this method are reverse-scored and linearly transformed from 0 to 100 (0 = 100 to 4 = 0), according to the following pattern: the higher the scores the better HRQL.

#### Psychometric properties

All of the methods, including the PedsQL 4.0 Generic Core Scales, the PedsQL 4.0 SF15 Generic Core Scales, and the PedsQL 3.0 Rheumatology Module have proven good reliability and validity. A study conducted on 963 pediatric patients and 1,629 parents, indicated internal consistency reliability for the Total Scale Score of the PedsQL 4.0 Generic Core Scales with Cronbach’s alpha values rated on 0.88 for a child self, and 0.90 for parent proxy report. The validity was demonstrated using the known-groups method and showed that this instrument differentiated healthy subjects from patients with acute or chronic diseases [60]. The Total Score, Physical Health Summary Score, and Psychosocial Health Summary Score from the PedsQL 4.0 SF15 were adequately reliable for all age group comparisons, with alpha coefficient > or = 0.70, as reported by Chan et al. The PedsQL 4.0 SF15 distinguished between children varying in health status [61]. The PedsQL 3.0 Rheumatology Module’s reliability, validity, and usefulness were also demonstrated elsewhere, based on a study of 231 children and 244 parents, followed in the pediatric rheumatology clinic. The investigators showed again excellent reliability for age group comparisons in which Cronbach’s alpha ranged from 0.75 to 0.86 for a child self-report, and from 0.82 to 0.91 for a parent proxy report. Validity was appointed by comparing groups of children with different clinical status (the known-groups method) and resulted in sufficient quality [59].

The PedsQL 4.0 Generic Core Scales and the PedsQL 3.0 Rheumatology Module have already been used in JIA [62–64], and also in other rheumatic diseases like lupus erythematosus [65] or fibromyalgia [66].

### Patient-reported outcomes measurement information system® (PROMIS®): a new possibility

#### Content

The US National Institutes of Health (NIH) Patient-Reported Outcomes Measurement Information System® (PROMIS®) was designed to evolve standardized instruments (short forms, item banks, computer adaptive tests (CATs) measuring multiple domains of physical, mental, and social health in children and adults [67–69]. The measurements are not disease-specific and allow assessment across many chronic conditions, treatment settings, and the general population [70].

The Pediatric Global Health (PGH-7) measure and its extended version (PGH-7 + 2) are dedicated to HRQL assessment in children. The PGH-7 comprises a seven-item brief summary of a child’s general, physical, mental, and social health. To each of the items belong five response options. The first four items’ response options correspond to grading scores of 5 down to 1, respectively. The fifth, sixth, and seventh items’ response options score through 5 to 1 for item number five and from 1 up to 5 for items number six and seven, respectively.

The extended version of the Pediatric Global Health (PGH-7 + 2) has incorporated two calibrated items from PROMIS® pediatric and parent proxy item banks to evaluate fatigue and pain interference, with response options scored from 1 up to 5, respectively [Supplementary material]. The recall period for these two items is 7 days.

#### Practical application

Both PGH-7 and PGH-7 + 2 can be administered as a patient self-report for children aged 8–17 years or as a parent proxy report for children aged 5–17 years. Both measures are fixed length short forms obtainable in paper/hard copy as well as in electronic formats on platforms for computer administration – Assessment Center API (including REDCap) and Epic PROMIS CAT Application. The PGH-7 has been affordable in 18 different language versions and the PGH-7 + 2 in 19 so far, whereas further consecutive translations are in preparation.

#### Scoring

The PGH-7 and the PGH-7 + 2 are able to generate global health scores, additionally, the PGH- 7 + 2 produces separate scores for pain interference and fatigue item. These two scores do not contribute to the global health score and are qualified as a preliminary estimate for pain interference and fatigue. Both measurements, as all of the PROMIS® domains, are scored with item-level calibrations based on Item Response Theory (IRT) – a group of statistical models. The IRT scores are transformed to the T-score metric and reported with a mean of 50 and a standard deviation of 10. The most accurate implementation of the scoring is the usage of the Health Measures Scoring Service or an electronic data collection tool, e.g. Assessment Center, REDCap auto-score. If such proceeding is not possible, the tables converting raw scores into T-score metric values are to be applied.

#### Psychometric properties

The PGH-7 measure’s internal consistency alpha coefficient was rated on 0.88 for the child self-report form and 0.84 for the parent proxy report form, as identified in the developmental study of 3635 children aged 8–17 years and 1,807 parents of children aged 5–17 years, conducted among US population. Both self and parent proxy reports showed excellent test- retest reliability. There was no invariance in item functioning by age, gender, race, or ethnicity. Furthermore, the study’s findings proved that the PGH-7 items were well understood by children from the age of 8 years upwards [71]. Another study on a group of 4636 American 8–17-years-old children and their 2609 parents showed convergent and discriminant validity of both the PGH-7 self-reported and parent proxy versions with PROMIS pediatric measures of physical, mental, and social health. Children with chronic conditions, Hispanic ethnicity, and those with low socioeconomic status generated lower scores of the PGH-7 measurement [72].

Moreover, the PGH-7 measure has already been trialed in several chronic conditions among the pediatric population, e.g. asthma [73], diabetes type 1, inflammatory bowel disease, cystic fibrosis [74, 75], celiac disease [76], nephrotic syndrome [77], cerebral palsy [78], and Down syndrome [79], not to mention recent attempts of evaluation of COVID-19 pandemic’s impact on the mental and social health of children and adolescents [80, 81]. Among asthmatic patients, the PGH-7 measure’s reliability based on Cronbach’s alpha oscillated from 0.66 to 0.81 for child self-report, and from 0.76 to 0.82 for parent proxy report. Patients with well- controlled asthma had PGH-7 scores higher than patients who were uncontrolled, with a conclusion that the PGH-7 was a reliable and valid assessment of general health status among children with asthma [73].

At the moment, numerous of the pediatric measures, e.g. anger, anxiety, depressive symptoms, fatigue, mobility, pain interference, pain behavior and peer relationships, have already been tested in JIA [35], still yet, the PGH-7 and the PGH-7 + 2 have not been successfully utilized in this disease. As so the PGH-7 has the potential to be a useful clinical and research tool assessing children's self or parent proxy reported global health status and quality of life, future research should focus on the implementation of this measure just in JIA.

## Discussion

This review described several currently available PROMs intended to measure HRQL, non-specific to JIA. PROMs are foreseen to be the future of healthcare management for both the adults and children [82], however, using PROMs in clinical care in children leads to some challenges. Principally, a disturbing issue is the construction of mentally- and age-adapted contents and formats [83, 84]. Nevertheless, it is proven that children from seven years of age, and sometimes younger, are capable of understanding and reporting to PROMs [85]. For younger children, or those unable to respond according to the health conditions, parental proxy reports are available as well. Still yet, as shown in the literature on pediatric health outcomes, compatibility between parent proxy and self-reports of HRQL may considerably differ. Results of parent proxy HRQL measurements are different depending on the methodology and instrument used [86]. Moreover, there is a tendency of parents of children with chronic diseases to evaluate their children’s HRQL as much poorer than it would be noted from the child’s perspective itself [87]. Thus, both the parent proxy and child self-reported outcomes have their pros and cons, still yet, whenever a child can respectively respond, the child’s self-report should always become an overall objective [88].

The majority of the presented PROMs are feasible in assessing HRQL in the pediatric population, both healthy children and those with chronic inflammatory diseases. However, all of the instruments differ in their properties and have specific limitations. One of the disparities between the instruments is score development. The PROMIS® PGH-7 and the EQ-5D-Y are unidimensional, generating one general health score/state. The PedsQL 4.0 Generic Core Scales and the PedsQL 3.0 Rheumatology Module are regarded as multidimensional, producing scales, one for each of the dimensions [Table 1]. At this point, there is a need to highlight that contrastingly to other abovementioned PROMs, the PedsQL instruments include disease-specific modules complementary to the general HRQL assessment, what constitutes an advantage in the care of chronically ill children, and allow to more precisely and adequately measure HRQL.

As reported elsewhere, self-evaluated patient’s HRQL state can importantly vary from the patient’s HRQL state reported by others [89], especially in the comparison of assessment conducted by an adult and by a child. The HRQL instruments should have child self- and parent proxy reports calibrated and validated separately, and, as proven, this requirement is fulfilled by the majority of the described PROMs – the PROMIS® PGH-7, the PedsQL 4.0 Generic Core Scales, the PedsQL 4.0 SF15 Generic Core Scales, the PedsQL 3.0 Rheumatology Module and the EQ-5D-Y. Still yet, the usage of the EQ-5D-Y meets an opportunity of the insufficient number of the specific child and adolescents value sets, as the great number of presently available value sets are based on the adult population [46].

Currently, the golden standard in disease-specific HRQL assessment in JIA is the Juvenile Arthritis Multidimensional Assessment Report (JAMAR), which consists of 15 dimensions combining general (physical function, pain-VAS scale, HRQL, overall well-being- VAS scale) and disease-specific outcomes (number of inflammated joints, morning stiffness, extra-articular symptoms, disease activity-VAS scale, actual disease status, disease course, treatment and its side effects). HRQL outcome includes two subscales, the Physical Health (5 items) and the Psychosocial Health (5 items), allowing total and separate scoring for both subscales. Both self- and parent proxy reports are accessible [36, 90].

The JAMAR gives a full view on both patient’s health status and course of the disease and is recommended to use whenever disease’s impact on the child’s well-being should be precisely rated, e.g. relapse, exacerbation [57]. The PedsQL 3.0 Rheumatology Module was created to complement the general HRQL assessment and may be insufficient to substitute disease-specific PROMs in such cases. However, when the disease-specific outcomes are not essentially needed, presented PROMs could be a better option. The EQ-5D-Y, the PedsQL 4.0 Generic Core Scales and the PedsQL 4.0 SF15 Generic Core Scales are more complex in the matter of dimensions and contained therein items, what can result in more comprehensive HRQL assessment. What is more, the EQ-5D-Y, and both the PedsQL 4.0 Generic Core Scales and the PedsQL3.0 Rheumatology Module, have already been tested in JIA, still yet, such validation and cross-cultural adaptation research need to be performed with the use of PROMIS® PGH-7 and PGH-7 + 2 measurements.

The PROMIS® PGH-7 and PGH-7 + 2 seem to be particularly promising in the healthcare of JIA patients, due to their properties, briefness (7/9 items) and connected with these procedures time-effectiveness, various forms of comfortable electronic administration, facile scoring, general availability, and optional integration with other PROMIS® pediatric measurements. By implication, this specific tool means an inevitable opportunity for future directions in JIA [45, 71].

## Conclusion

Using the HRQL questionnaires in pediatric rheumatology is evidently below expectations. This may be due to limited awareness of health care professionals, whereas attitudes of physicians towards patient perspectives seem to be underachieved. The PROMs reflect most accurately patient perception of the disease and are valuable components of the holistic medical care, although the evaluation methods used may be time-consuming and challenging while interpretation in children may implicate difficulties [83, 84]. Therefore, these methods have not yet been brought into general use, and have been underrated. The child’s voice and opinions, in particular those based on the PROMs, play an important role and may offer an interesting opportunity to extend practical knowledge, leading thereby to the improvement of long-term rheumatologic care and the patient-physician relationship. Even if the implementation of PROMs in routine check-ups is difficult on a regular basis, these methods would add practical value and would benefit the appropriate management of JIA [89]. In conclusion, the PROMs may provide a standardized tool for a better insight into the psychosocial nature of the chronic pediatric disease, and for understanding possible poor clinical outcomes, monitoring disease severity, and response to treatment.

## Supplementary Information

Below is the link to the electronic supplementary material.

Supplementary file1 (DOCX 15 KB)

## Data Availability

The raw data supporting the conclusions of this article will be made available by the authors, without undue reservation, the medical history of the patients.

## Abbreviations

EQ-5D: EuroQol
HRQL: Health-related quality of life
JIA: Juvenile idiopathic arthritis
PedsQL: Pediatric quality of life inventory
PGH: Pediatric global health
PROMIS: Patient-reported outcomes measurement information system
PROMs: Patient-reported outcome measures
QoL: Quality of life
