# Supplementary material for: Non-disease specific patient-reported outcome measures of health-related quality of life in juvenile idiopathic arthritis: a systematic review of current research and practice
Source: Rheumatol Int. 2021 Dec 31;42(2):191–203. doi: 10.1007/s00296-021-05077-x (PMC8719533; doi:10.1007/s00296-021-05077-x)
Supplement: Supplementary file 1 — Supplementary file1 (DOCX 15 KB) [file 296_2021_5077_MOESM1_ESM.docx]

**Supplementary material
RHEI-D-21-01344 R1**

**Table.** Linguistic characteristics, detailed response options and exact wording of the available PROMS.

| Instrument / Method |  | Wording |
| --- | --- | --- |
| EQ-5D-3L | Severity levels | „no problems”, „some problems”, „extreme problems” |
|  | The most severe option in mobility dimension | „confined to bed” |
|  | The most severe option in self-care dimension | „being unable to” |
|  | The most severe option in usual activities dimension | „being unable to” |
|  | The most severe option in pain/discomfort dimension | „I have an extreme pain or discomfort” |
|  | The most severe optio in anxiety/depression dimension | „I am extremely anxious or depressed” |
|  | The mildest option in self-care dimension | „I have no problems with self-care” |
| EQ-5D-5L | Severity levels | „no problems”, „slight problems”, „moderate problems”, „severe problems” and „unable to/extreme problems” |
|  | The most severe option in mobility dimension | „unable to walkabout” |
| EQ-5D-Y | Severity levels | „no problems/no pain/not worried”, „some problems/some pain/a bit worried” and „a lot of problems/a lot of pain/very worried” |
|  | The most severe option in mobility dimension | „a lot of problems walking about” |
|  | The most severe option in looking after myself dimension | „having a lot of problems” |
|  | The most severe option in doing usual activities dimension | „having a lot of problems” |
|  | The most severe option in having pain or discomfort dimension | „I have a lot of pain or discomfort” |
|  | The most severe option in feeling worried, sad or unhappy dimension | „I am very worried, sad or unhappy” |
|  | The mildest option in looking after myself dimension | „I have no problems washing or dressing” |
| EQ-VAS | Endpoints | „The best health you can imagine” and „The worst health you can imagine” |
| PROMIS® Pediatric Global Health (PGH-7) | Response options | „excellent”, „very good”, „good”, „fair”, „poor” for the first four items and „never”, „rarely”, „sometimes”, „often”, „always” for the remaining items |
|  | Item 1 | „In general, would you say your health is:…” |
|  | Item 2 | „In general, would you say your quality of life is:…” |
|  | Item 3 | „In general, how would you rate your physical health?” |
|  | Item 4 | „In general, how would you rate your mental health, including your mood and ability to think?” |
|  | Item 5 | „How often do you feel really sad?” |
|  | Item 6 | „How often do you have fun with friends?” |
|  | Item 7 | „How often do your parents listen to your ideas?” |
| PROMIS® Pediatric Global Health (PGH-7+2) | Response options for fatigue and pain interference item | „never”, „almost never”, „sometimes”, „often”, „almost always” |
|  | Fatigue item | „I got tired easily.” |
|  | Pain interference item | „I had trouble sleeping when I had pain.” |
